# Duodenal metastasis of hepatocellular carcinoma following multimodal therapy: a rare case report and literature review

**DOI:** 10.1007/s13691-026-00872-4

**Published:** 2026-05-13

**Authors:** Nobuhisa Tanioka, Satoru Seo, Kohei Araki, Kazune Fujisawa, Masaya Munekage, Hiromichi Maeda, Hiroyuki Kitagawa, Tsunehiro Ochi, Mitsunari Ogasawara, Akira Hirose

**Affiliations:** 1 Department of Surgery, Kochi Medical School Hospital, 185-1 Kohasu, Okocho, Nankoku-city, Kochi 783-8505 Japan; 2 Department of Gastroenterology and Hepatology, Kochi Medical School Hospital, Nankoku-city, Kochi 783-8505 Japan

**Keywords:** Hepatocellular carcinoma, Duodenum, Metastasis

## Abstract

We present a rare case of duodenal metastasis of hepatocellular carcinoma (HCC) following multimodal therapy, in which the patient died shortly after the palliative surgical intervention. An 80-year-old man with HCC associated with chronic hepatitis B was admitted to our emergency department owing to complaints of anorexia and vomiting. He had undergone conversion surgery following systemic therapy that included a multikinase inhibitor and immune checkpoint inhibitors. Three months prior, transcatheter arterial chemoembolization and radiofrequency ablation had been performed for two intrahepatic recurrences. Contrast-enhanced abdominal computed tomography and upper gastrointestinal endoscopy revealed multiple intrahepatic recurrences, pulmonary metastases, and duodenal metastasis of HCC. Following the resolution of obstructive jaundice, the patient underwent palliative gastrojejunostomy. His postoperative course was complicated by progressive renal dysfunction and coagulopathy, and he died on postoperative day 15. In the literature review, including this case, 61 patients with duodenal involvement of HCC were identified. Although a small subset of patients achieved long-term survival after curative-intent surgery performed under highly selective conditions, the overall life expectancy for most patients remained poor. Even with palliative intent, surgical intervention for unresectable duodenal involvement caused by HCC should be pursued with careful consideration of the patient’s prognosis and systemic condition.

## Introduction

Hepatocellular carcinoma (HCC) is the most common primary malignant tumor of the liver. Despite declines in incidence and mortality in many high-risk regions owing to the development of antiviral agents and advances in multimodal therapy, HCC remains the third leading cause of cancer-related death worldwide, following lung and colorectal cancers [1].

HCC usually disseminates within the liver. Extrahepatic metastases occur in 30–50% of cases, most commonly to the lungs, followed by the bones and lymph nodes [2, 3]. In contrast, gastrointestinal (GI) involvement is rare, with a reported incidence of 0.5–2% [4]. Among these, direct invasion of the duodenum by HCC has been frequently reported, whereas hematogenous or lymph node metastasis is extremely uncommon.

Here, we report a case of HCC that developed duodenal metastasis following multimodal therapy, including immune checkpoint inhibitors and conversion surgery, and resulted in early death after palliative surgical intervention.

## Case report

An 80-year-old man with chronic hepatitis B-related HCC presented to our emergency department with progressive anorexia and vomiting. Three years prior, he was diagnosed with a 7.5-cm multinodular confluent-type HCC occupying hepatic segments 6 and 7. Although he received atezolizumab plus bevacizumab for two months, the disease progressed. Subsequently, lenvatinib was administered for 11 months with the intent of tumor downsizing, followed by durvalumab plus tremelimumab for two months. Because portal vein thrombosis developed during the course, anticoagulation therapy was initiated and the regimen was switched to transcatheter arterial infusion using cisplatin with lenvatinib. The portal vein thrombosis dissolved, and treatment with the same regimen was continued for 10 months; however, cisplatin had to be discontinued due to the development of acute kidney injury. Consequently, right hepatectomy was performed as a conversion surgery one year prior to presentation. Five months before admission, two intrahepatic recurrent lesions in segments 2/3 and 4 were identified and treated with lenvatinib. Three months prior, transarterial chemoembolization and radiofrequency ablation were performed.

**Fig. 1:**
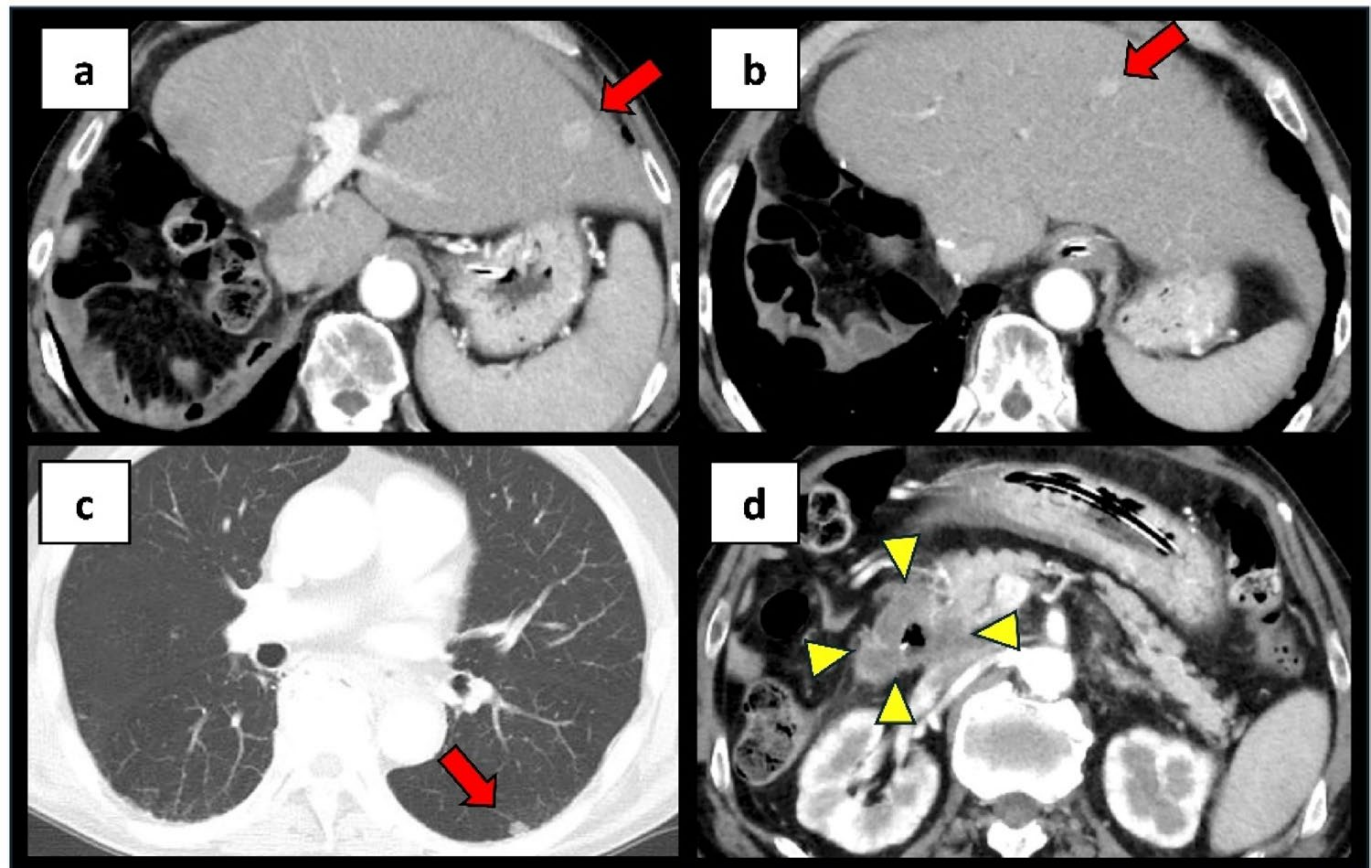
Contrast-enhanced computed tomography at presentation. Two nodular lesions are observed in the left hepatic lobe (arrows) and a solitary nodule is seen in the left lower lobe of the lung (arrow) (**a–c**). In addition, circumferential wall thickening of the descending duodenum is observed (arrowheads) (**d**)

**Fig. 2:**
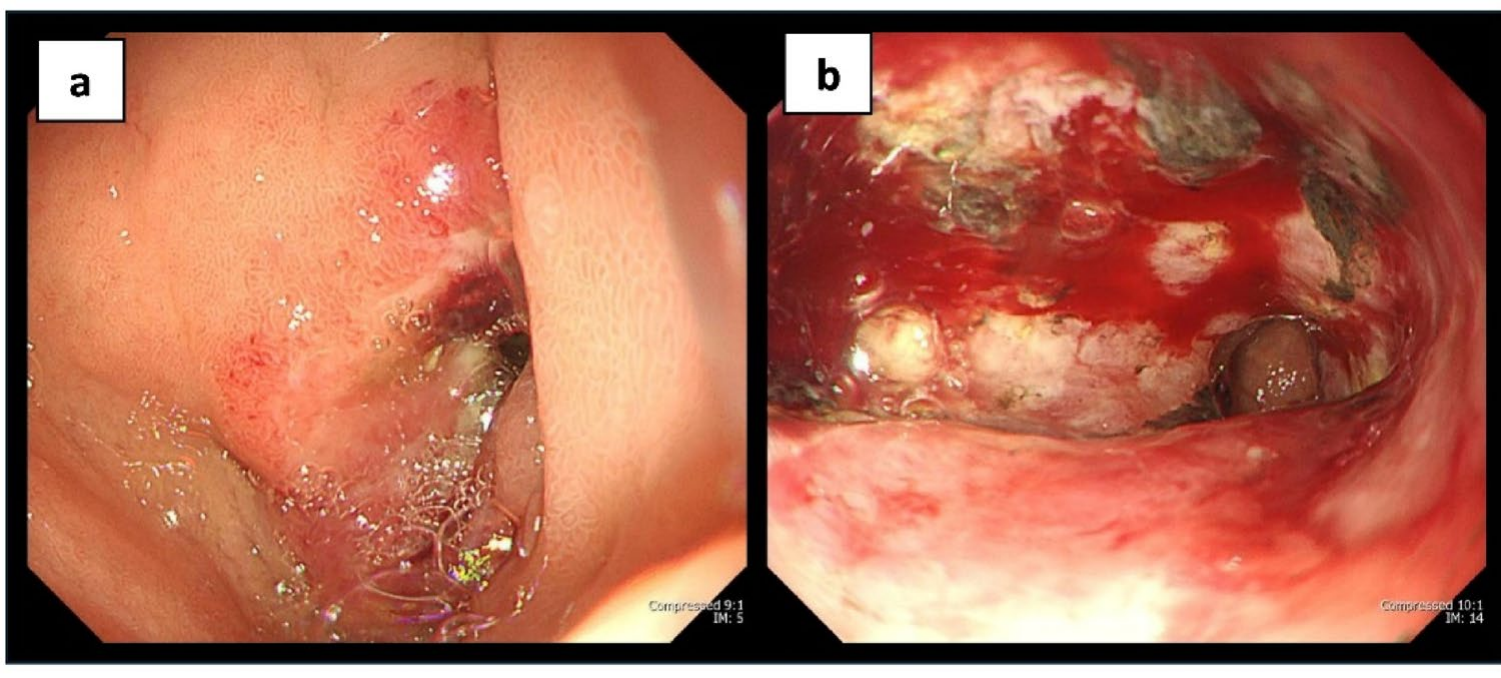
Upper gastrointestinal endoscopy after admission demonstrates retained food in the stomach and a 4-cm ulcerative, elevated lesion in the descending duodenum proximal to the papilla

**Fig. 3:**
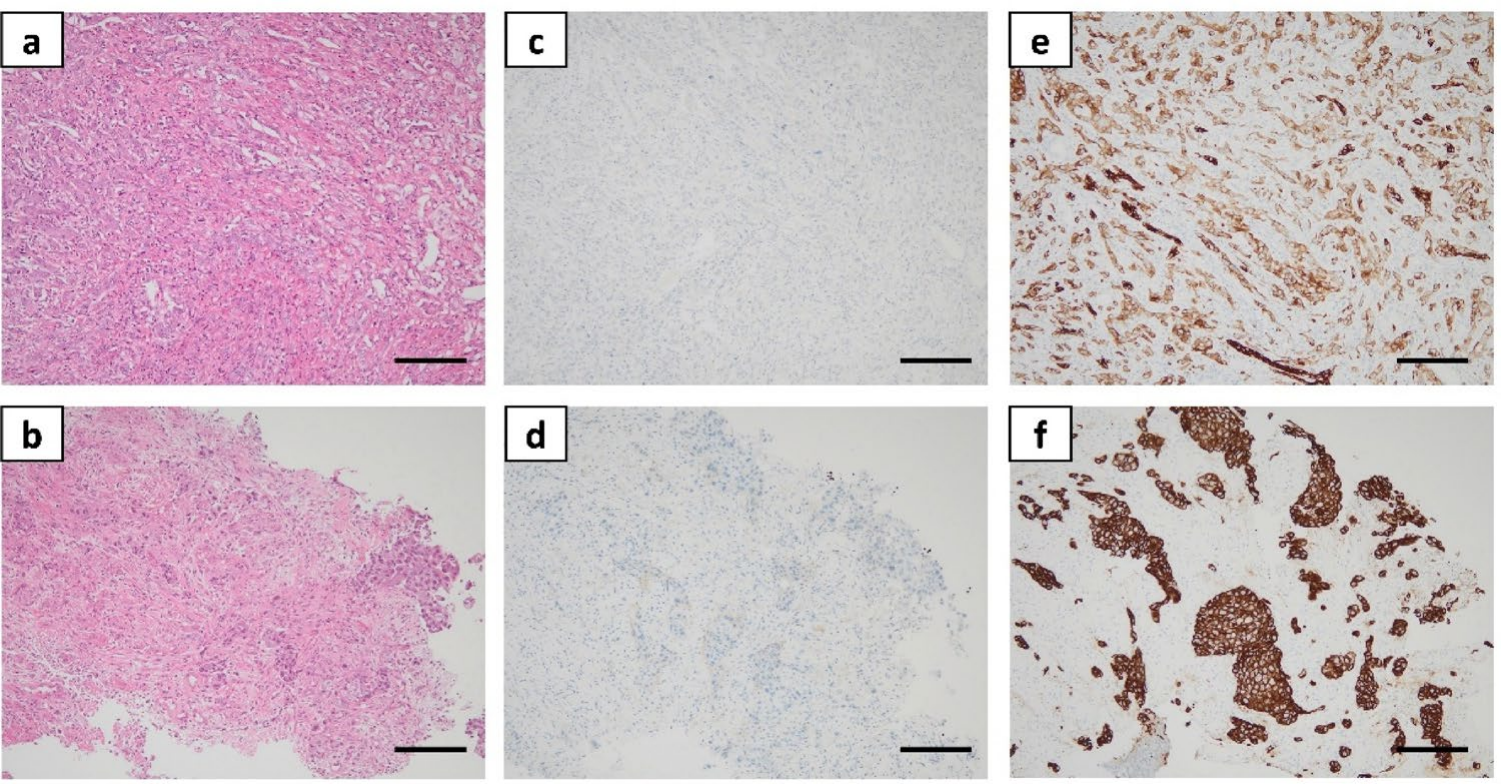
Histopathological and immunohistochemical comparison between the primary hepatocellular carcinoma and the duodenal lesion. (**a**) Hematoxylin and eosin staining of the primary tumor demonstrating poorly differentiated hepatocellular carcinoma. (**b**) Hematoxylin and eosin staining of the duodenal biopsy specimen demonstrating undifferentiated carcinoma. (**c**) Immunohistochemical staining for hepatocyte marker showing negative staining in the primary tumor. (**d**) Immunohistochemical staining for hepatocyte marker in the duodenal biopsy specimen showing negative staining. (**e**) Immunohistochemical staining for CK7 demonstrating positive tumor cells in the primary tumor (**f**) Immunohistochemical staining for CK7 demonstrating positive tumor cells in the duodenal biopsy specimen. Scale bar = 200 μm (a–f)

At presentation, laboratory findings revealed a hemoglobin level of 8.1 g/dL, a platelet count of 120 × 10³/µL, total bilirubin of 1.6 mg/dL, aspartate aminotransferase of 59 U/L, alanine aminotransferase of 49 U/L, alpha-fetoprotein of 89.4 ng/mL, which remained relatively low and fluctuated after surgery, and protein induced by vitamin K absence-II of 241 U/mL, which showed a persistent increase following surgery. Contrast-enhanced computed tomography revealed two new nodules in the left hepatic lobe, a pulmonary nodule in the left lower lobe, and circumferential wall thickening of the descending duodenum (Fig. 1). Upper GI endoscopy revealed retained food in the stomach and a 4-cm ulcerative, elevated lesion in the descending duodenum proximal to the papilla (Fig. 2). Biopsy revealed undifferentiated carcinoma. Immunohistochemical analysis showed negative staining for hepatocyte antigen and positive staining for CK7. Because the resected liver specimen had been diagnosed as poorly differentiated HCC with an identical immunohistochemical profile, the lesion was diagnosed as duodenal metastasis from HCC (Fig. 3). Nutritional support was initiated via enteral feeding. On hospital day 7, the patient developed obstructive jaundice due to biliary invasion and underwent percutaneous transhepatic biliary drainage. Although biliary drainage and nutritional support were provided, the patient’s condition remained poor, with a prognostic nutritional index of 23.9, a Glasgow Prognostic Score of 2, Eastern Cooperative Oncology Group performance status of 3, and American Society of Anesthesiologists Physical Status of 3. To enable oral intake, a palliative gastrojejunostomy was performed on hospital day 37. No peritoneal dissemination was observed intraoperatively. The blood loss was minimal, and the operative time was 105 min.

**Fig. 4:**
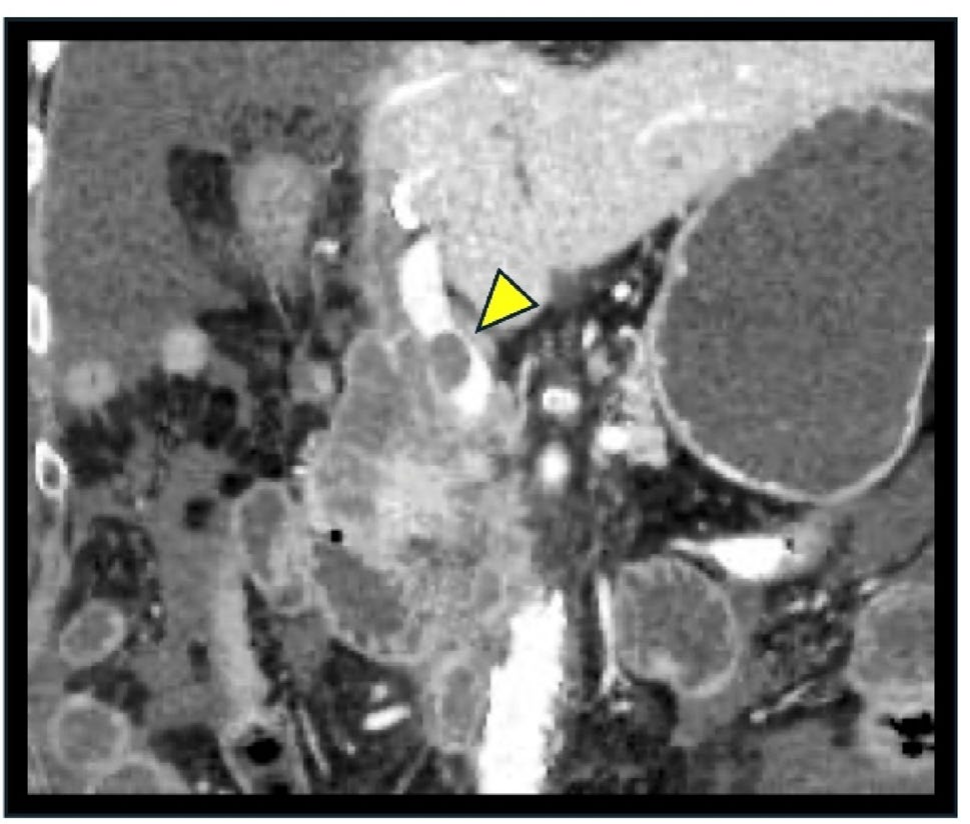
Contrast-enhanced computed tomography reveals massive ascites and portal vein thrombosis on postoperative day 6 (arrowhead)

**Fig. 5:**
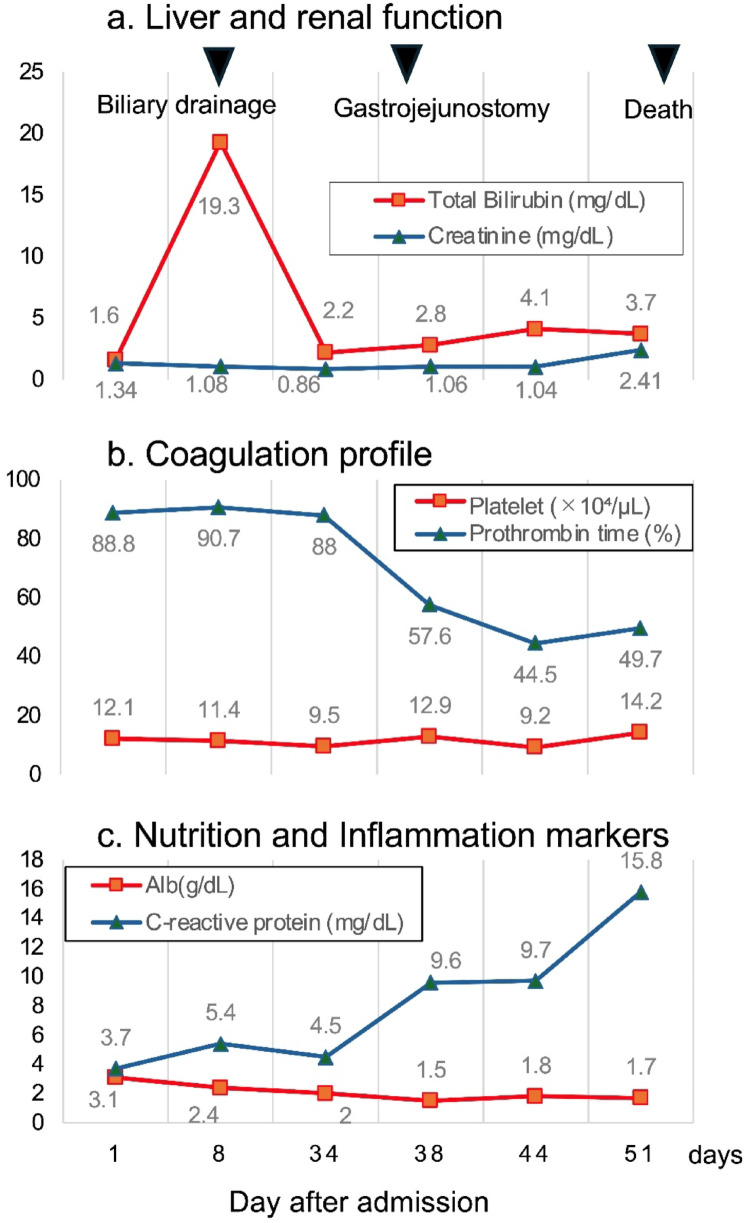
Temporal changes in laboratory parameters after admission, presented in subpanels A–C. Liver and renal function (**a**), coagulation profile (**b**), and nutritional and inflammatory markers (**c**)

Immediately after surgery, the patient developed hypoalbuminemia and oliguria, and albumin supplementation together with diuretic therapy was initiated. As coagulation markers showed progressive worsening, contrast-enhanced CT was performed on postoperative day 6 and demonstrated portal vein thrombosis (Fig. 4). Anticoagulation therapy was started, both renal function and coagulation status continued to deteriorate (Fig. 5). On postoperative day 13, a transition to best supportive care was decided, and all aggressive interventions were discontinued. The patient died on postoperative day 15.

## Discussion

GI involvement in HCC is rare and is sometimes identified as one of the multiple lesions in end-stage HCC with peritoneal dissemination or extrahepatic metastases [2]. Moreover, because the duodenum and stomach are anatomically adjacent to the liver, they represent sites that are particularly susceptible to direct invasion by HCC [5]. Such cases typically involve large tumors with aggressive invasive potential, indicating an advanced stage [6]. In this case, the simultaneous detection of lung metastases and the absence of disseminated lesions outside the duodenum during surgery led to clinical suspicion of hematogenous duodenal metastasis.

The mechanisms of hematogenous metastasis to the duodenum remain unclear. However, several possibilities can be postulated. First, portal hypertension may play a role; irrespective of liver cirrhosis, major hepatic resections are known to result in postoperative splenomegaly [7], suggesting portal venous stasis or retrograde flow. Second, transarterial chemoembolization (TACE) and systemic therapies may promote the growth and dissemination of aggressive tumor clones by upregulating angiogenic factors or selecting for resistant subclones [8]. Third, although no studies have directly investigated this issue, hepatic resection and TACE may promote the development of collateral circulation and a relative increase in arterial inflow to the pancreaticoduodenal region, potentially facilitating hematogenous metastasis to this area [9].

Importantly, the duodenum possesses unique anatomical features, including the confluence of the bile and pancreatic ducts, necessitating a distinct clinical and therapeutic approach compared with that of other GI sites. A PubMed search using “duodenal metastases” and “duodenal involvement” of HCC as keywords yielded 30 publications. These studies, together with our case, involved 61 patients and are summarized in Table 1 [4, 6, 10–37]. The mean patient age was 62.6 ± 8.2 (range, 34 − 83) years, 86.8% were male. The most common route of involvement was direct invasion in 43 patients, followed by lymphatic or hematogenous metastases in 6, hematogenous metastases in 3, peritoneal dissemination in 2, and lymph node metastases in 1. Portal vein thrombosis was observed in 12 patients. At the time of diagnosis, 44 patients (72.1%) had received some form of prior treatment, regardless of whether duodenal involvement was due to direct invasion or metastasis (70.5% vs. 68.6%). A history of immune checkpoint inhibitor therapy was observed only in our case.

**Table 1:** Literature review of cases with duodenal involvement of hepatocellular carcinoma

First Author	Year	Age	Sex	Route of involvement	Maximum liver tumor size (cm)	Other extrahepatic metastasis	PVT	Previous treatment for HCC	Duodenal involvement treatment	Surgical procedure	Survival after diagnosis (Months)
Humbert [10]	1987	70	M	D	13.0	−	−	−	None		0.3
Chen [11]	1990	56	M	D	22.0	−	NA	−	Surgery	Gastrojejunostomy	0.8
		56	M	D	6.0	−	−	TAE	TAE		1.0
		54	M	D	8.0	−	+	TAE, RT	IA chemo		4.0
		34	M	D	NA	−	NA	Chemo	None		2.0
Arima [12]	1992	61	M	H	3.0	−	+	SR, Chemo	Supportive		17
Moriura [13]	1995	57	M	D	7.0	−	−	−	Surgery	Major hepatectomy with partial duodenectomy	22
Okusaka [14]	1997	60	M	D	11.0	−	−	SR, TAE, PEI	Supportive		Post-mortem diagnosis
Hung [15]	1998	58	M	D	4.0	−	−	SR, TAE, Chemo	RT		6.0
Farrell [16]	1999	53	M	D	8.0	−	−	SR	Supportive		NA
Srivastava [17]	2000	48	M	D	NA	−	−	−	TACE		2.0
Lin [4]	2000	64	M	H	10.0	−	+	−	Supportive		2.2
		67	M	D	15.0	−	+	−	NA		1.5
		56	M	D	12.0	−	+	−	NA		3.0
Del Natale [18]	2001	67	M	D	NA	−	+	TACE	NA		NA
Cho [19]	2002	50	M	D	22.0	−	−	−	Surgery	Major hepatectomy with partial duodenectomy	NA
Ohnishi [20]	2004	73	M	D	9.0	−	−	SR, TAE, RFA, PEI, RT	TAE		2.0
Uehara [21]	2003	62	M	L	1.0	LN	−	−	Surgery	Partial hepatectomy with LN dissection	22.0
Hung [22]	2008	67	M	NA	None	−	−	LT, TACE	NA		NA
Chung [23]	2009	53	F	NA	NA	−	+	−	NA		7.0
Kurtz [24]	2009	78	F	D	8.5	−	−	RFA, sorafenib	NA		NA
Kato [25]	2010	63	M	D	25.0, 2.0	−	−	−	Surgery	Major HPD	8.0
Lin [26]	2011	72	M	D	4.5	−	−	PEI, RAE, SR	Surgery	Partial hepatectomy with distal gastrectomy	68.0
Liang [6]	2012	Median 62.5	M: 17 F: 4	D	8.3	LN	−	−: 4, SR: 4, SR+TACE: 7, SR+TACE + PEI: 1, TACE + RFA: 1, TACE: 4, TACE + PEI: 1	Supportive		1.1
				D	9.6	−	−		Surgery	NA	1.9
				D	12.0	Adrenal gland, LN	+		Supportive		1.2
				H/L	13.0	−	−		Supportive		1.1
				H/L	5.0	−	−		Surgery	NA	0.3
				D	5.0	−	−		TACE		2.0
				D	5.0	−	−		Supportive		8.8
				D	7.8	−	−		Supportive		14.4
				NA	NA	NA	−		Supportive		31
				D	6.0	LN	−		Surgery	NA	24.8
				D	12.0	LN	+		Supportive		5.3
				D	7.5	Bone	−		TACE		6.9
				H/L	None	Peritoneum	−		Surgery	NA	2.7
				D	8.3	Adrenal gland, lung	−		Supportive		1
				D	12.0	Stomach, IVC	+		Supportive		8.6
				H/L	None	Colon	−		Surgery	NA	57.8
				H/L	2.0	−	−		Surgery	NA	5.2
				D	8.0	Adrenal gland, lung	−		Supportive		2.5
				D	10.0	−	−		Supportive		0.2
				D	14.0	−	−		RT		42.7
				H/L	9.5	LN	−		Supportive		0.2
Kim [27]	2012	57	M	D	NA	−	+	TACE	Supportive		3.0
Sauer [28]	2012	68	M	D	NA	−	NA	TACE, RT, Chemo	Supportive		1.0
Lee [29]	2021	59	M	D	NA	NA	NA	TACE, PEI	Supportive		1.0
		72	M	NA	NA	NA	NA	TACE, PEI	Supportive		0.3
		50	M	NA	NA	NA	NA	SR, Chemo	Supportive		3.0
		64	M	NA	NA	NA	NA	TACE, RT	Supportive		1.0
		62	M	D	NA	NA	NA	TACE, PEI	Supportive		1.0
		49	M	D	NA	NA	NA	TACE, PEI	Supportive		1.0
Arima [30]	2015	76	F	P	6.0	−	−	SR	Surgery	Partial duodenectomy	NA
Kashani [31]	2015	62	M	P	NA	−	−	TACE	Supportive		3.0
Lin [32]	2018	83	M	D	NA	−	NA	TACE	NA		NA
Ito [33]	2019	65	M	D	10.0	−	−	TACE, sorafenib	Surgery	Major hepatectomy with partial duodenectomy	36.0
Liu [34]	2020	62	M	D	2.4	−	−	RFA, SR	Surgery	Partial duodenectomy and gastrojejunostomy	84.0
Wu [35]	2020	80	F	D	25	−	NA	−	NA		NA
Bonboire [36]	2021	67	M	D	7.0	−	−	−	Sorafenib		6.0
Sawada [37]	2021	72	M	D	NA	−	−	TACE	TAE		7.5
Tanioka N.	2025	80	M	H	2.0	Lung	+	Ate/Bev, Dur/Tre, Lenvatinib, IA chemo, TACE, RFA, SR,	Surgery	Gastrojejunostomy	6.0

PVT: portal vein thrombosis; HCC: hepatocellular carcinoma; F: female; M: male; NA: not available; D: direct invasion; H: hematogenous metastasis; L: lymphatic metastasis; H/L: hematogenous or lymphatic metastasis; P: peritoneal dissemination; LN: lymph node; IVC: inferior vena cava; TAE: transarterial embolization; RT: radiation therapy; Chemo: Chemotherapy; SR: surgical resection; RFA: radiofrequency ablation; PEI: percutaneous ethanol injection; RAE: radiation arterial embolization; LT: liver transplantation; TACE: transarterial chemoembolization; IA chemo: intrahepatic arterial infusion chemotherapy; Ate/Bev: atezolizumab plus bevacizumab; Dur/Tre: durvalumab plus tremelimumab; HPD: hepatopancreatoduodenectomy

Excluding 8 cases with unavailable treatment data, management strategies for duodenal involvement included supportive care in 25 patients (47.2%), curative-intent surgery in 8 (15.1%), gastrojejunostomy in 2 (3.8%), TACE or transarterial embolization in 6 (11.3%), radiotherapy in 2 (3.8%), sorafenib in 1 (1.9%), and intra-arterial infusion chemotherapy in 1 (1.9%). The overall median survival was 3.0 months (range, 0.2–84). The median survival was 2.8 months in the direct invasion group and 4.0 months in the hematogenous or lymph node metastasis group (Mann–Whitney U test, *p* = 0.89). These outcomes were markedly shorter than the reported overall survival of patients with distant metastasis of HCC (OS, 17.6 months) [38]. In contrast, patients who underwent curative-intent surgery (*n* = 8) demonstrated substantially longer survival than those managed with non-surgical treatments (*n* = 9), with median survivals of 29.0 months (range, 8–84) and 4.0 months (range, 1–42.7), respectively. Curative surgical procedures included partial duodenal resection, distal gastrectomy, lymphadenectomy, and hepatopancreatoduodenectomy. Accordingly, curative resection is recommended for patients without other factors precluding resectability and with an acceptable operative risk.

In our case, the patient had multiple intrahepatic metastases and pulmonary metastases in addition to duodenal metastasis, and his overall condition was compromised by malnutrition and obstructive jaundice. Although a palliative gastrojejunostomy was performed to enable oral intake, he developed portal vein thrombosis due to progressive coagulopathy and died in the early postoperative period. Given the patient’s preoperative general condition and anticipated prognosis, a less invasive palliative approach should have been selected.

Endoscopic duodenal stent placement for malignant duodenal obstruction provides a clinical success rate comparable to that of gastrojejunostomy, despite a relatively high rate of re-obstruction [39]. Accordingly, it is considered a suitable option for patients with poor prognosis and compromised performance status. Lee et al. reported 6 cases of HCC with duodenal involvement treated with self-expandable metallic stents [29]. Obstructive jaundice occurred in 1 patient, whereas 5 patients were able to resume at least a soft diet, and stent patency was maintained for a mean of 51 days (range, 10–119).

Multimodal therapy for HCC has expanded with the advent of immune checkpoint inhibitors, resulting in improved survival outcomes [40, 41]. As an increasing number of patients proceed to subsequent lines of therapy, uncommon metastatic patterns, such as those observed in the present case, are likely to be encountered more frequently. Accordingly, further accumulation of evidence regarding the clinical features and treatment outcomes of these cases is warranted.

In conclusion, because the prognosis of unresectable duodenal involvement from HCC is markedly poor, palliative surgical interventions should be undertaken with careful consideration of the patient’s overall condition.
